# Bioinformatics screening the novel and promising targets of curcumin in hepatocellular carcinoma chemotherapy and prognosis

**DOI:** 10.1186/s12906-021-03487-9

**Published:** 2022-01-25

**Authors:** Tingting Yang, Yibiao Chen, Jiexuan Xu, Jinyuan Li, Hong Liu, Naihua Liu

**Affiliations:** 1 Scientific Research Center, The First Affiliated Hospital of Guangdong Pharmaceutical University, Gonghexiheng Street 1, Guangzhou, Guangdong 510080 P.R. China; 2 Department of Head and Neck Radiotherapy, Meizhou City People’s Hospital, No.6 Building, Huangtang Road 63, Meijiang District, Meizhou, Guangdong 514031 P.R. China; 3 Department of Traditional Chinese Medicine, The First Affiliated Hospital of Guangdong Pharmaceutical University, Gonghexiheng Street 1, Guangzhou, Guangdong 510080 P.R. China; 4 Key Specialty of Clinical Pharmacy, The First Affiliated Hospital of Guangdong Pharmaceutical University, Nonglin Down Street 19, Guangzhou, Guangdong 510080 P.R. China

**Keywords:** Bioinformatics analysis, Hepatocellular carcinoma, Curcumin, Chemotherapy, Prognosis

## Abstract

**Background:**

The aim of present study was to screen the novel and promising targets of curcumin in hepatocellular carcinoma diagnosis and chemotherapy.

**Methods:**

Potential targets of curcumin were screened from SwissTargetPrediction, ParmMapper and drugbank databases. Potential aberrant genes of hepatocellular carcinoma were screened from Genecards databases. Fifty paired hepatocellular carcinoma patients’ gene expression profiles from the GEO database were used to test potential targets of curcumin. Besides, GO analysis, KEGG pathway enrichment analysis and PPI network construction were used to explore the underlying mechanism of candidate hub genes. ROC analysis and Kaplan-Meier analysis were used to evaluate the diagnostic and prognostic value of candidate hub genes, respectively. Real-time PCR was used to verify the results of bioinformatics analysis.

**Results:**

Bioinformatics analysis results suggested that *AURKA*, *CDK1*, *CCNB1*, *TOP2A*, *CYP2B6*, *CYP2C9*, and *CYP3A4* genes served as candidate hub genes. *AURKA*, *CDK1*, *CCNB1* and *TOP2A* were significantly upregulated and correlated with poor prognosis in hepatocellular carcinoma, AUC values of which were 95.7, 96.9, 98.1 and 96.1% respectively. There was not significant correlation between the expression of *CYP2B6* and prognosis of hepatocellular carcinoma, while *CYP2C9* and *CYP3A4* genes were significantly downregulated and correlated with poor prognosis in hepatocellular carcinoma. AUC values of *CYP2B6*, *CYP2C9*, and *CYP3A4* were 96.0, 97.0 and 88.0% respectively. In vitro, we further confirmed that curcumin significantly downregulated the expression of *AURKA*, *CDK1*, and *TOP2A* genes, while significantly upregulated the expression of *CYP2B6*, *CYP2C9*, and *CYP3A4* genes.

**Conclusions:**

Our results provided a novel panel of *AURKA*, *CDK1*, *TOP2A*, *CYP2C9*, and *CYP3A4* candidate genes for curcumin related chemotherapy of hepatocellular carcinoma.

**Supplementary Information:**

The online version contains supplementary material available at 10.1186/s12906-021-03487-9.

## Background

Recently, the incidences of live cancer ranked fifth and ninth in male and female cancer, respectively. Its mortality ranked second and sixth in male and female cancer, respectively [1]. Besides, according to global cancer statistics 2018, China shared 46.7% of global liver cancer cases [2]. Hepatocellular carcinoma serves as the most common type of primary liver cancer, with its high-risk factors at least including metabolic liver disease, hepatitis virus infection, and alcohol abuse [3, 4]. Long-term exposure of hepatitis B/C virus would develop chronic viral hepatitis, then followed by liver cirrhosis and hepatocellular carcinoma [3, 5]. Nonalcoholic fatty liver disease or metabolic associated fatty liver disease, one typical metabolic liver disease, would develop nonalcoholic steatohepatitis, then followed liver cirrhosis and hepatocellular carcinoma [6, 7]. Alcohol abuse would induce chronic liver injury, which developed liver fibrosis and eventually progressed to hepatocellular carcinoma [8].

Earlier-stage clinical symptoms of hepatocellular carcinoma were vague or nonspecific, thus hepatocellular carcinoma patients usually were diagnosis at an intermediate and advanced stage. Surgical resection was the ideal option for earlier- stage hepatocellular carcinoma patients without cirrhosis, while transplantation was the best option for those earlier- stage hepatocellular carcinoma patients with cirrhosis [9, 10]. Systemic therapies at least include chemotherapy, immunotherapy and radiotherapy, which were strongly recommended for hepatocellular carcinoma patients at intermediate and advanced stage. However, up to date, chemoprevention and adjuvant therapy regarded as much less efficient interventions in advanced hepatocellular carcinoma treatments [9, 11].

Natural herb or peptide have exhibited antioxidant, anti-inflammatory, and anti-proliferative effects on disease treatment [12–16]. Accumulating evidence indicated that curcumin was a promising natural compound, which has been extensively investigated and shown multiply therapeutic activities, at least including anticancer, anti-virus, anti-arthritis, anti-amyloid, anti-oxidation, and anti-inflammatory [17]. In the molecular events of liver disease, curcumin inhibits HBV gene expression and replication via down-regulation of PGC-1α [18]. Curcumin had no effect on HCV RNA replication or viral assembly/release, but impaired virus binding and entry into human liver cells [19]. Randomized Controlled Trials showed that curcumin significantly ameliorated nonalcoholic fatty liver disease [20, 21]. Curcumin increased PPARγ to inhibit the expression of SREBP-2 and low-density lipoprotein receptor, which subsequently inactivated hepatic stellate cells, curcumin also increased SREBP-1c to promote lipid storage [22]. Thus, these findings indicated the potential therapeutic value of curcumin in protecting against liver steatosis and fibrosis. Curcumin would decrease the stemness of liver cancer stem cells by attenuating NF-κB/HDAC signaling [23]. Interestingly, curcumin suppressed stromal cell-derived factor-1/CXCR4 signaling to reduce the incidence of circulating gastric cancer cells, and subsequently decreased the risk of secondary liver cancer [24]. Thus, these previous studies highlighted that curcumin might exhibit multifunction in the initiation and progression of liver cancer.

In the present study, we performed bioinformatic analysis to screen the targets of curcumin, which would contribute to initiation and progression of hepatocellular carcinoma. Then we further validated these candidates with gene profiles of hepatocellular carcinoma patients, and tried to answer the underlying mechanism.

## Methods

### Screening the potential curcumin-related targets for hepatocellular carcinoma therapy

Simplified Molecular Input Line Entry Specification (SMILES) structure of curcumin was obtained from Pubchem website (https://pubchem.ncbi.nlm.nih.gov/). The SMILES structure of curcumin was used to predict the potential *Homo Sapiens* target from SwissTargetPrediction database and PharmMapper database (version 2017) [25, 26]. We used “curcumin” to screen its verified targets from DrugBank database [27]. Curcumin relative targets were then integrated with the above three databases and removed the repeats. We used “Hepatocellular Carcinoma” and “Hepatocellular Cancer” items to acquire the potential hepatocellular carcinoma relative target from GeneCards database [28], then we transformed their gene ID from Uniprot database (https://www.uniprot.org/) for further analysis. We next merged the curcumin relative target and the hepatocellular carcinoma relative target and picked up the overlapped candidate for further analysis.

### Clinical data collection and processing

The hepatocellular carcinoma relative microarray data (GSE14520) was based on GPL3921 platform and downloaded from Gene Expression Omnibus (GEO) database by using GEOquery package (version 2.56.0) [29], the data was firstly normalized by using normalizeBetweenArrays function of limma package (version 3.44.3) [30–32]. We then obtained 50 fully paired gene expression matrix of normal adjacent liver tissues and hepatocellular carcinoma tissues from GSE14520. The principal component analysis (PCA) was performed to visually present the data by FactoMineR package (version 2.3) and factoextra package (version 1.0.7) [33]. The gene expression matrix and differential expression analysis were carried out by using pheatmap package (version 1.0.12).

### Gene functional annotations

The Kyoto Encyclopedia of Genes and Genomes (KEGG) pathway enrichment analysis was performed by using ClusterProfiler package (version 3.16.1) and visualized by using RColorBrewer package (version1.1-2) [34–36]. The Gene Ontology (GO) enrichment analysis, including Biological process (BP) analysis, Cellular component (CC) analysis, and Molecular function (MF) analysis, were performed by using ClusterProfiler package (version 3.16.1) [37], ggplot2 package (version 3.3.3), and stringr package (version 1.4.0). *P*-value less than 0.05 was regarded as the cutoff value of statistical significance.

### Protein-protein interaction (PPI) network construction

The selected candidate gene was uploaded onto Search Tool for the Retrieval of Interacting Genes (STRING, version 11.0) databaseto predict and construct a potential PPI network [38], then network data integration, analysis, and visualization were performed by using Cytoscape software (version 3.7.1).

### Expression, prognosis, and diagnosis analysis of selected genes

In order to track the dynamic expression of selected genes in initiation and progression of hepatocellular carcinoma, we downloaded the mRNA expression profile and the corresponding clinical information of hepatocellular carcinoma from The Cancer Genome Atlas (TCGA) database (http://cancergenome.nih.gov/) by TCGAbiolinks package (version 2.16.3), including normal samples (*n* = 44), stage I samples (*n* = 175), stage II samples (*n* = 86), stage III samples (*n* = 84), and stage IV samples (*n* = 5). Expression data of selected genes were processed by GraphPad Prism 9, and analyzed by Student’s t-test. The Kaplan-Meier survival analysis was constructed by Gene Expression Profiling Interactive Analysis (GEPIA) on-line tool [39]. The receiver operating characteristic (ROC) curve was plotted by using dplyr package (version 1.0.3) and pROC package (1.16.2) [40]. *P*-value less than 0.05 was regarded as the cutoff value of statistical significance.

### Cell culture and treatment

HepG2.2.15 cell line was derived from HepG2 cell which was transfected full length DNA of Hepatitis B Virus, purchased from China Center for Type Culture Collection, and which was a gift from Dr. Liufeng Mao. For cell culture, HepG2.2.15 cells were maintained in complete growth Dulbecco’s Modified Eagle Medium (DMEM) containing 4.5 g/L glucose (Life Technologies, Inc., Carlsbad, CA), 10% fetal bovine serum, 100 U/ml penicillin, and 100 μg/ml streptomycin. All cells were incubated at 37 °C with 5% CO2. For cell treatment, HepG2.2.15 cells were seeded into 12-well plates at 2 × 10^5^ cells/well overnight, then cells were treated with 1, 4, 10 μM curcumin (CSNpharm, China) for further 24 h, dimethyl sulfoxide (Beyotime, China) was used as the vehicle control.

### RNA isolation and quantitative RT-PCR

Total RNA was extracted using the RNAeasy™ Animal RNA Isolation Kit with Spin Column (Beyotime, China) according to manufacturer’s instructions. Total RNA was reversely transcribed amplified using the BeyoFast™ SYBR Green One-Step qRT-PCR Kit (Beyotime, China). All real-time PCR primers were listed as following: *AURKA*, 5′-CTAACGGCTGAGCTCTTGGA-3′ and 5′-GAACCGACAGGGGACTTGAC-3′. *CCNB1*, 5′-ACCTTTGCACTTCCTTCGGA-3′ and 5′-TGTTCTTGACAGTCCATTCACCA-3′. *CDK1*, 5′-GCCCTTTAGCGCGGATCTAC-3′ and 5′-AGGAACCCCTTCCTCTTCACT-3′. *TOP2A*, 5′-CCGTCACCATGGAAGTGTCA-3′ and 5′-TGTCTGGGCGGAGCAAAATA-3′. *CYP2B6*, 5′-CCTCAACCTCAACACGCTCT-3′ and 5′-TTTGGCTCGGTCATGAAGCT-3′. *CYP2C9*, 5′-ACCAGCTGTGCTTCATTCCT-3′ and 5′-GCACAGTGAAACATAGGAAACTCTC-3′. *CYP3A4*, 5′-GCTTTCCTGCACATTAAGGAGAA AT-3′ and 5′-ATGGGCAAAGTCACAGTGGAT-3′. *GAPDH*, 5′-AGCCTCAAGATCATCAGC-3′ and 5′-GAGTCCTTCCACGATACC-3′. Relative mRNA expression levels of target genes were normalized by comparing to *GAPDH*, and calculated using the 2 − ΔΔCt method [41]. Expression data of selected genes were processed by GraphPad Prism 9, and analyzed by Student’s t-test. *P*-value less than 0.05 was regarded as the cutoff value of statistical significance.

### Cell counting kit-8 assay

Cells were seeded into 96-well plates at 10000 cells/well, after culture for 24 h, cells were treated with indicated concentrations of curcumin (CSNpharm, China) for further 48 h, and then the cell vitality was measured by the cell counting kit-8 (Beyotime, China) kit. The cellular viability (%) = OD value (treated cell) / OD value (control cell) × 100%.

## Results

### Potential targets of curcumin in hepatocellular carcinoma therapy

**Fig. 1:**
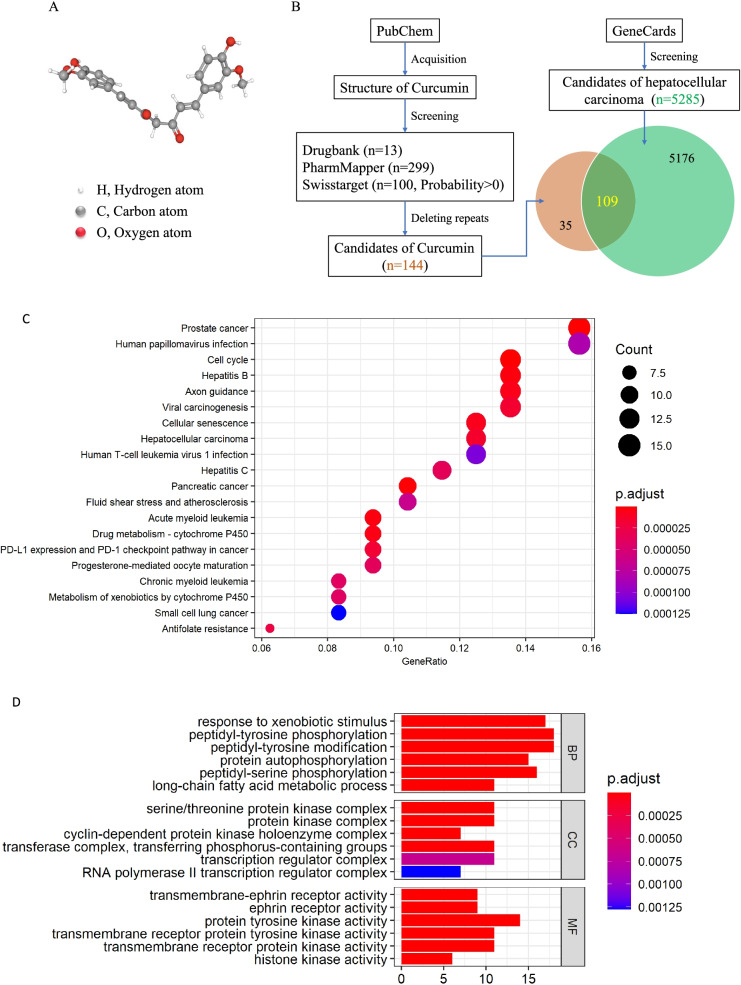
Screening curcumin-related targets in hepatocellular carcinoma therapy. **A** 3D conformer of curcumin (acquired by PubChem). **B** Workflow of Screening curcumin-related targets in hepatocellular carcinoma therapy. **C** KEGG pathway analysis of 109 selected potential targets. **D** Biological process (BP), Cellular component (CC), and Molecular function (MF) of GO analysis of 109 selected potential targets

Canonical SMILES of curcumin is COC1 = C(C=CC(=C1)C=CC(=O)CC(=O)C=CC2 = CC (=C(C=C2)O)OC)O (Computed by OEChem 2.1.5, PubChem release). 3D conformer of curcumin was shown in Fig. 1A. Thirteen candidates of curcumin were acquired by Drugbank, 299 candidates of curcumin were acquired by PhamMapper, 100 candidates (Probability> 0) of curcumin were acquired by Swisstarget (Fig. 1B). After deleting the repeats candidates, we obtained 144 candidates of curcumin (Fig. 1B). Five thousand two hundred eighty-five aberrant genes were acquired by GeneCards, when they overlapped with candidates of curcumin, we here obtained 109 potential targets of curcumin in hepatocellular carcinoma therapy (Fig. 1B). KEGG pathway analysis showed that aberrant expression of these 109 potential targets significantly and mainly correlated with viral-induced carcinoma, at least including prostate cancer, hepatocellular carcinoma, pancreatic cancer, and leukemia. They also highly correlated with cytochrome P450-related drug metabolism (Fig. 1C). Biological process (BP) of GO analysis showed that these 109 potential targets mainly regulated xenobiotic-induced protein phosphorylation in long-chain fatty acid metabolic process (Fig. 1D). Cellular component (CC) of GO analysis showed that these 109 potential targets mainly regulated activities of protein kinases in the transcriptional process (Fig. 1D). Molecular function (MF) of GO analysis showed that these 109 potential targets mainly responded to activities of the transmembrane receptor protein kinase and the histone kinase (Fig. 1D).

### Validating the 109 selected potential targets in GSE14520 dataset

**Fig. 2:**
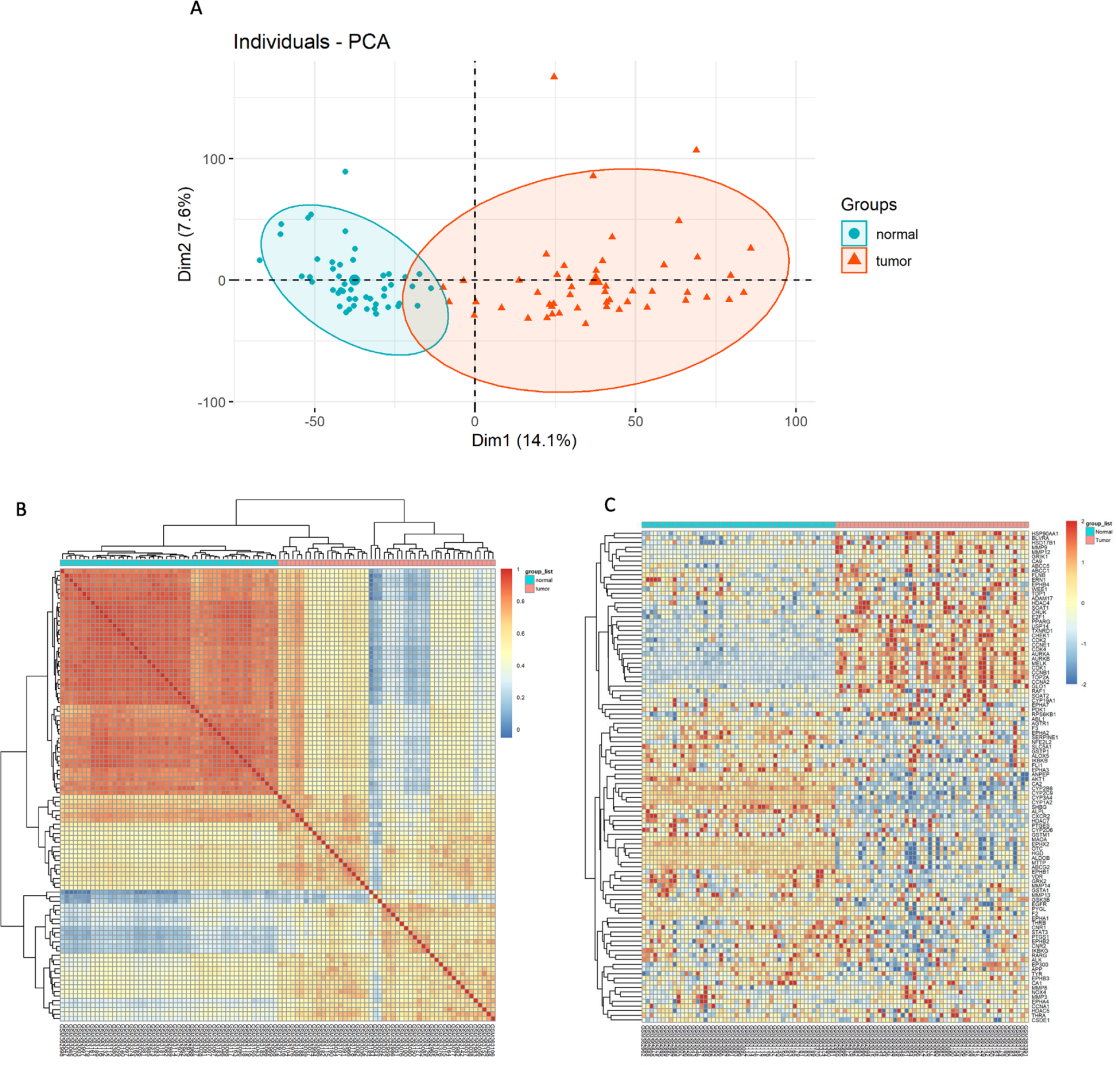
Identification of the 109 selected potential targets in GSE14520 dataset. **A** Principal component analysis of the GSE14520 dataset, each turquoise dot represented a normal adjacent liver tissue, and each tangerine triangle represented a hepatocellular carcinoma tissue. **B** Correlation coefficient heatmap analysis of the GSE14520 dataset. **C** Expression of 106 selected potential targets in the GSE14520 dataset

Gene profiles of 50 paired normal adjacent liver tissues and hepatocellular carcinoma tissues were extracted from GSE14520 dataset. Principal component analysis (PCA) showed that there was a clear distinction between normal adjacent liver tissues and hepatocellular carcinoma tissues (Fig. 2A). Correlation coefficient heatmap analysis also showed that there was a significant difference of gene profiles between the two groups. Besides, Genetic variation in normal adjacent liver tissues was smaller than those in hepatocellular carcinoma tissues (Fig. 2B). Among these 109 selected potential targets, 3 selected potential targets (*BRAF*, *TLR9*, *CDK3*) were missing in the GSE14520 dataset. 9 (8.5%) selected potential targets were significantly upregulated in GSE14520 array, 14 (13.2%) selected potential targets were significantly downregulated in GSE14520 dataset, the rest had no significant difference (Fig. 2C).

### PPI network analysis and functional annotations of the selected potential targets

**Fig. 3:**
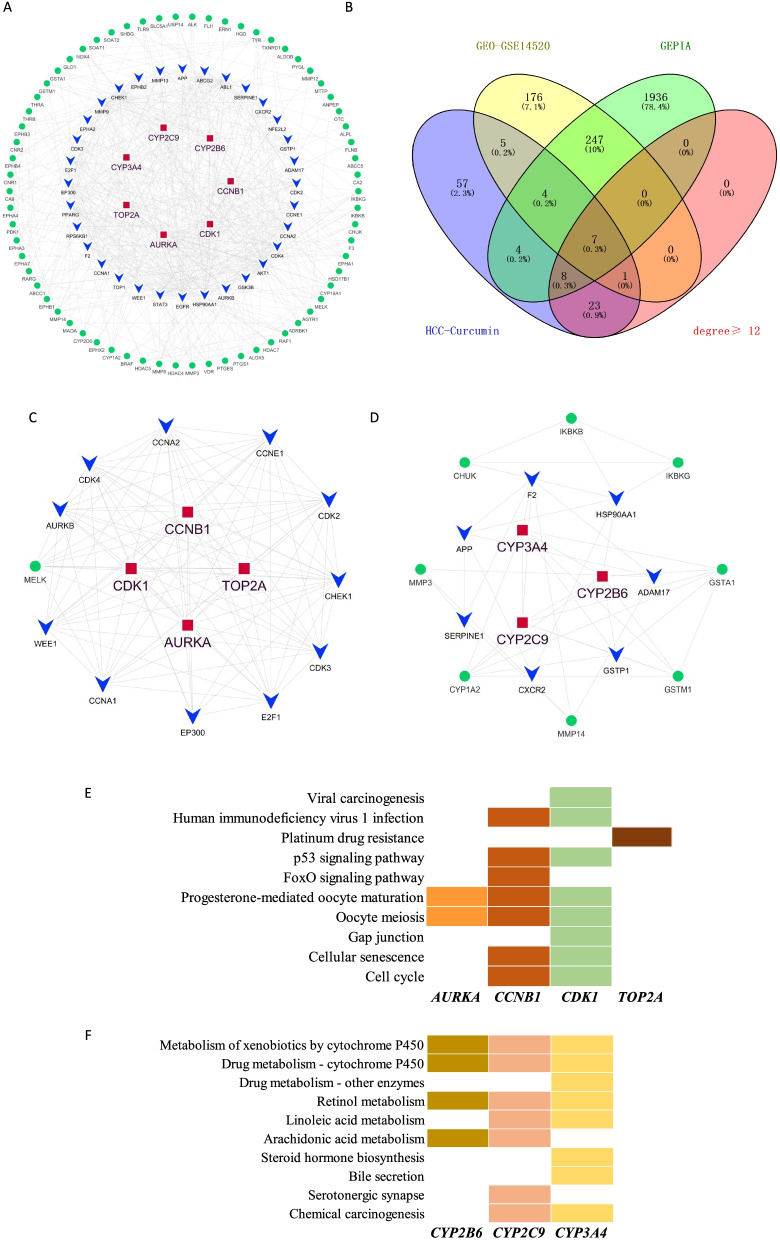
PPI network construction and functional annotations of the selected potential targets. **A** PPI network construction of the 109 selected potential targets, the red square represented the communal gene (Degree distribution≥12), the blue arrow represented the hub gene that degree distribution was greater than or equal 12, the green dot represented the hub gene that degree distribution was less than 12. **B** Venn diagram showed that seven communal genes were found among these indicated datasets. **C** Core network construction of *AURKA*, *CCNB1*, *CDK1* and *TOP2A*. **D** Core network construction of *CYP2B6*, *CYP2C9,* and *CYP3A4*. **E** KEGG pathway analysis of *AURKA*, *CCNB1*, *CDK1* and *TOP2A*. **F** KEGG pathway analysis of *CYP2B6*, *CYP2C9,* and *CYP3A4*

According to PPI network analysis, 105 nodes were found, and the average number of neighbors (degree distribution) was 11.505 (Fig. 3A, Supplemental Table 1). Besides, the top list of hub nodes (Degree distribution>11) were showed in Supplemental Table 2. When we merged these hub genes with differentially expressed genes in GSE14520 dataset and differentially expressed genes of hepatocellular carcinoma in GEPIA database, seven communal genes were found, they were *AURKA*, *CDK1*, *CCNB1*, *TOP2A*, *CYP3A4*, *CYP2C9*, and *CYP2B6* (Fig. 3B). We then constructed the core-network of these seven hub genes and their neighbor genes (Fig. 3C and D). Our results further showed that *AURKA*, *CCNB1* and *CDK1* involved in Oocyte meiosis and maturation via regulated FoxO signaling and/or p53 signaling. Besides, *CCNB1* and *CDK1* were also involved in virus-related carcinogenesis. *TOP2A* was mainly involved in platinum drug resistance (Fig. 3E). *CYP3A4*, *CYP2C9*, and *CYP2B6* mainly involved in cytochrome P450 related metabolism under physiological or pathological situations (Fig. 3F).

### Clinical correlation of selected genes in hepatocellular carcinoma

**Fig. 4:**
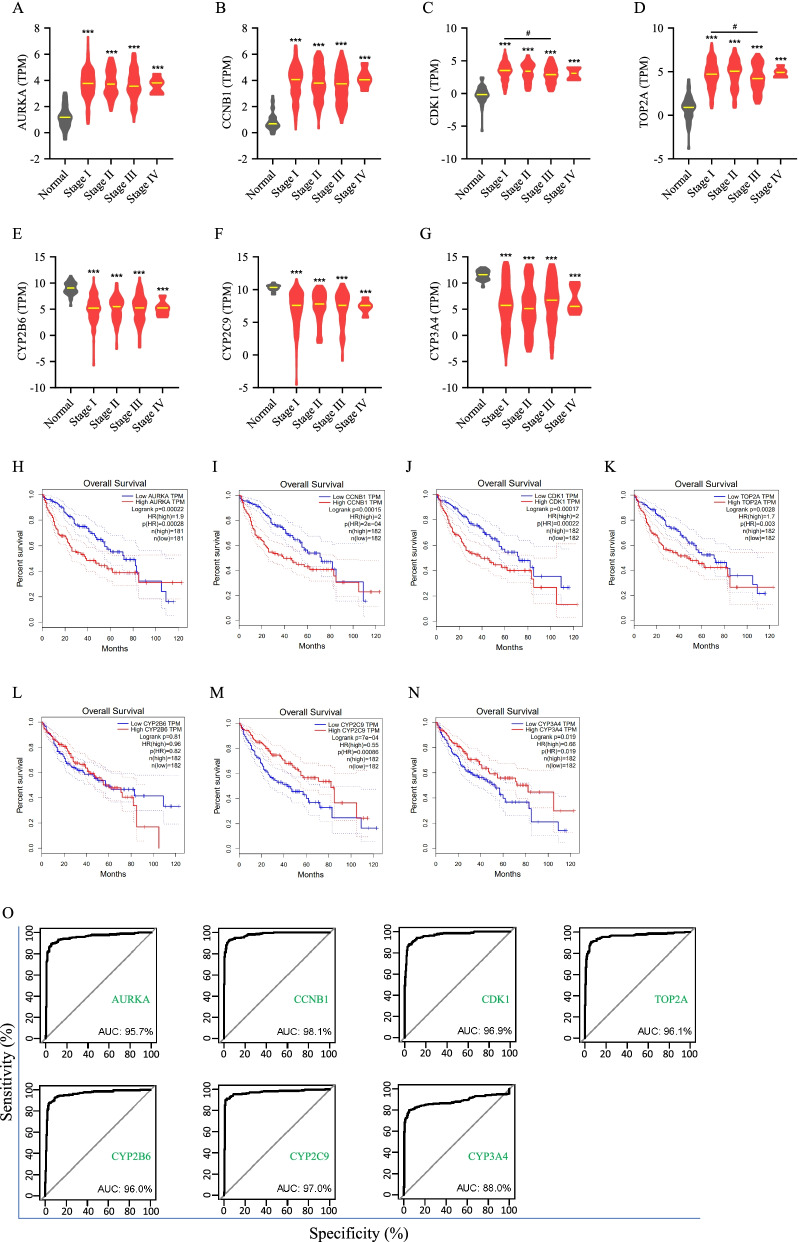
Expression, prognosis, and diagnosis of selected genes in hepatocellular carcinoma. **A**-**G** The mRNA expression of *AURKA*, *CCNB1*, *CDK1*, *TOP2A*, *CYP2B6*, *CYP2C9* and *CYP3A4* in hepatocellular carcinoma patients’ tumor tissues and adjacent normal tissues. **H**-**N** Kaplan-Meier analysis of Overall Survival for hepatocellular carcinoma patients based on the expression of *AURKA*, *CCNB1*, *CDK1*, *TOP2A*, *CYP2B6*, *CYP2C9* and *CYP3A4*. **O** ROC curve analysis for *AURKA*, *CCNB1*, *CDK1*, *TOP2A*, *CYP2B6*, *CYP2C9* and *CYP3A4* in hepatocellular carcinoma. ***p < 0.001, tumor tissues of each stage versus adjacent normal tissues; ^###^*p* < 0.001 tumor tissues of stage I versus tumor tissues of stage III

Transcriptional expression profile of hepatocellular carcinoma patients’ tumor tissues and adjacent normal tissues was extracted from TCGA database. The expression of *AURKA*, *CCNB1*, *CDK1*, or *TOP2A* in each stage samples was higher than the expression of its normal samples. There was not significantly difference of expression of *AURKA*, *CCNB1*, *CDK1*, or *TOP2A* between any two different stage samples, except for the expression of *CDK1* and *TOP2A* in stage III samples were slightly and significantly decreased while compared with stage I samples (Fig. 4A-D). The expression of *CYP2B6*, *CYP2C9*, or *CYP3A4* in each stage samples were less than the expression of its normal samples. There was not significantly difference of expression of *CYP2B6*, *CYP2C9*, or *CYP3A4* between any two different stage samples (Fig. 4E-G). Besides, *AURKA*, *CCNB1*, *CDK1* and *TOP2A* positively and significantly correlated with poor prognosis of hepatocellular carcinoma, while *CYP2C9* and *CYP3A4* negatively and significantly correlated with poor prognosis of hepatocellular carcinoma, but there was not significant correlation between the expression of *CYP2B6* and prognosis of hepatocellular carcinoma (Fig. 4H-N). ROC curves showed that the AUC of *AURKA*, *CCNB1*, *CDK1*, *TOP2A*, *CYP2B6*, *CYP2C9* and *CYP3A4* were 95.7, 98.1, 96.9, 96.1, 96.0, 97.0 and 88.0%, respectively (Fig. 4O).

### The effect of curcumin on regulating the target genes

**Fig. 5:**
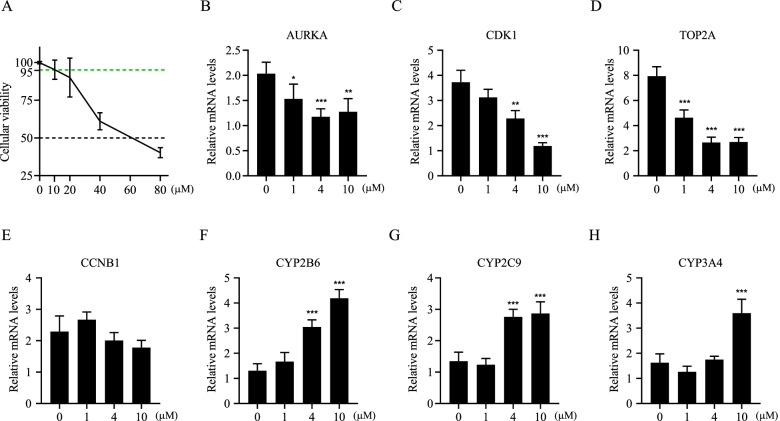
The effect of curcumin on regulating the target genes. **A** After treating with a series concentration of curcumin for 48 h, the cellular viability of HepG2.2.15 cells were measured by CCK8 assay. **B**-**H** After treating HepG2.2.15 cells with a series concentration of curcumin for 24 h, mRNA expression of *AURKA*, *CDK1*, *TOP2A*, *CCNB1*, *CYP2B6*, *CYP2C9,* and *CYP3A4* were analyzed by real-time PCR, *GAPDH* served as housekeeping gene

We next investigated the effect of curcumin on regulating the target genes. Our results showed that curcumin dose-dependently decreased cellular viability in HepG2.2.15 cells, and the cellular viability remained at least 95% when the concentration of curcumin was under or equal to 10 μM (Fig. 5A). The lower dose (from 0 μM to 10 μM) of curcumin was then used for the subsequent experiments. Compared to the vehicle treatment, curcumin significantly decreased mRNA expression of *AURKA*, *CDK1*, and *TOP2A* (Fig. 5B-D). Curcumin had no effect on transcription of *CCNB1* (Fig. 5E). Besides, higher concentrations of curcumin significantly increased mRNA expression of *CYP2B6*, *CYP2C9*, and *CYP3A4* (Fig. 5F-H).

## Discussion

Liver take predominantly advantage in multi-fundamental physiological processes, at least including material metabolism, digestion, detoxification, and coagulation. Carcinoma in liver not only defected its bio-functions, but also attenuated clinical treatments and amplified the toxic and side-effects [9]. As described above, accumulating in vitro and in vivo studies have indicated that curcumin acted as a promising compound to exhibit multifunction in the prevention and treatment of liver cancer. We herein mainly focused and predicted the potential clinical effects of curcumin in hepatocellular carcinoma treatment. In the present study, we then screened and verified several novel and promising curcumin-target genes in hepatocellular carcinoma therapy via bioinformatics analysis approach.

Previous studies have showed that natural herb, at least including saffron, safranal, salvadora persica, ginger and their metabolites suppressed inflammatory, proliferative, and oxidative pathways, while triggered caspases activities and DNA instability to induced cytotoxicity and apoptosis in the liver cancer cells [42–46]. In the present study, seven common target genes were selected when we overlapped curcumin predicted targets, aberrant genes in initiation and progression of hepatocellular carcinoma, and differentially expressed genes of GSE14520 dataset. Previous studies showed that *AURKA* promoted proliferation and metastasis of hepatocellular carcinoma cells [47–49]. Besides, *AURKA* also involved in formation of secondary liver cancer [50, 51]. Blocking CDK1/PDK1/β-Catenin signaling would inhibit proliferation and EMT of hepatocellular carcinoma cells [52]. Previous studies showed that *CCNB1* was upregulated to promote proliferation of hepatocellular carcinoma via decreased its negative regulators, at least including RNA-binding motif protein 43 (RBM43), miR-199a-3p, or miR-144 [53–55]. So far as we know, only one study showed that synthetic resveratrol-curcumin hybrid compound 4c significantly decreased the expression *AURKA*, *AURKB*, and *CCNB1* in MCF-7 cells, it also significantly inhibited proliferation of MCF-7 cells, A549 cells, and HepG2 cells [56]. Otherwise, accumulating studies have shown that curcumin and its analogs significantly downregulated the expression of *CDK1* to induce cell cycle arrest in various human cancers, but not in hepatocellular carcinoma [57–59]. In the present study, our results showed that curcumin significantly decreased the transcription of *AURKA* and *CDK1* in HBV-transfected HepG2.2.15 cells, but not *CCNB1*. Otherwise, previous studies reported the dose of curcumin-induced cytotoxicity in HepG2 cells seemed to be contradictory. Lee and his college reported that no cytotoxicity was observed in HepG2 cells when curcumin was not more than 20 μM for 48 h treatment [60]. But Soni and his college reported that 10 μM curcumin sufficiently and significantly decreased the cell viability of HepG2 cells [61]. In line with Lee’ s result, our results showed that the lower dose (from 0 μM to 10 μM) curcumin has no significant effect on decreasing cellular viability of HepG2.2 cells. Taken these together, our results suggested that the lower dose curcumin treatment might not induced sufficient downregulation of *AURKA* or *CDK1* to inhibit proliferation of hepatocellular carcinoma cells. Alternatively, curcumin-induced downregulation of *AURKA* and *CDK1* might confer the other antitumor effects, but not inhibiting proliferation..

Previous studies showed that inhibition of *AURKA* would activate NF-κB signaling pathway to confer radio-resistance or chemoresistance in hepatocellular carcinoma and acute myeloid leukemia [62–64]. Blocking CDK1/PDK1/β-Catenin signaling would decrease stemness of cancer stem cells of hepatocellular carcinoma cells, and reverse sorafenib chemoresistance [52]. It’s reported that *TOP2A* was elevated in doxorubicin-resistant hepatocellular carcinoma cells, in line with this finding, the other group showed that *TOP2A* was elevated in doxorubicin-resistant hepatocellular carcinoma patients, *TOP2A* inhibitor etoposide would facilitate doxorubicin-induced cytotoxicity in primary cancer cells of hepatocellular carcinoma [65, 66]. *TOP2A* also conferred platinum resistance in several human cancers [67, 68]. In the present study, our results also showed that curcumin significantly decreased the transcription of *TOP2A* in HBV-transfected HepG2.2.15 cells. Accumulating studies have shown that curcumin exhibited therapeutic roles by facilitating the cytotoxicity of chemotherapeutic drugs and reversing their chemoresistance [69–73]. Thus, our results indicated that curcumin might decrease the expression of *AURKA*, *CDK1* and *TOP2A* to reverse chemoresistance in hepatocellular carcinoma treatment.

Cytochrome P450 (CYP) enzymes play important roles in endogenous and xenobiotic metabolism of liver, but their roles in tumorigenesis and progression remain as a complex context [74]. Previous studies have highlighted that most CYP members, such as *CYP2C9* and *CYP3A4*, were defected in hepatocellular carcinoma initiation and progression [75, 76]. *CYP2C9* was mainly activated in the metabolism drugs, at least including the activation of cyclophosphamide and tamoxifen, and the clearance of idarubicin [74]. *CYP3A4* was activated in the metabolism of procarcinogens, and contributed to the clearance of several chemotherapeutic agents, at least including cisplatin, etoposide or doxorubicin [74, 77]. *CYP2B6* also correlated with metabolism of procarcinogens, but it tended to involved in the metabolic activation of anticancer prodrugs [74]. In the present study, our results further showed that curcumin significantly upregulated the expression of CYP2B6, *CYP2C9* and *CYP3A4*. Otherwise, Previous studies have highlighted that curcumin ameliorated side-effects of chemotherapeutic drugs and exhibited hepatoprotective effects [20–22, 73]. Thus, the finding of us and the other group together suggested that it should pay more attention to curcumin-induced P450s on regulating the drug activation, inactivation or clearance while consumed with other certain anticancer drugs.

## Conclusion

In summary, so far as we known, our results first showed that *CDK1*, *TOP2A*, *CYP2C9,* and *CYP3A4* genes correlated to curcumin-related chemotherapy of hepatocellular carcinoma, more than just correlated to diagnosis and prognosis of hepatocellular carcinoma which had highlighted by the previous studies [76, 78]. Our result also showed that *AURKA* served as a new diagnosis and prognosis of hepatocellular carcinoma, even as also a potential and novel curcumin-related therapeutic target for hepatocellular carcinoma. Taken these together, the present study provided a new insight and proposal for curcumin-related hepatocellular carcinoma therapies.

## Supplementary Information

**Additional file 1: Supplemental Table 1.** Simple parameters of PPI network analysis. **Supplemental Table 2.** The top list of hub nodes in PPI network (Degree distribution≥12).

## Data Availability

The datasets analysed during the current study available from the corresponding author on reasonable request.

## Abbreviations

AURKA: Serine/Threonine-Protein Kinase Aurora-A
CDK1: Cyclin-Dependent Kinase 1
CCNB1: G2/Mitotic-Specific Cyclin-B1
TOP2A: DNA Topoisomerase 2-Alpha
CYP2B6: Cytochrome P450 2B6
CYP2C9: Cytochrome P450 2C9
CYP3A4: Cytochrome P450 3A4
AUC: Area Under the Curve
ROC: Receiver operating characteristic
HBV: Hepatitis B Virus
PGC-1α: Peroxisome Proliferative Activated Receptor Gamma Coactivator 1 Alpha
HCV: Hepatitis C Virus
PPARγ: Peroxisome Proliferator-Activated Receptor Gamma
SREBP-2: Sterol Regulatory Element-Binding Protein 2
NF-κB: Nuclear Factor Kappa-B
HDAC: Histone Deacetylase
CXCR4: Chemokine CXC Motif Receptor 4
SMILES: Simplified Molecular Input Line Entry Specification
GEO: Gene Expression Omnibus
KEGG: Kyoto Encyclopedia of Genes and Genomes
GO: Gene Ontology
BP: Biological process
MF: Molecular function
CC: Cellular component
STRING: Search Tool for the Retrieval of Interacting Genes
TCGA: The Cancer Genome Atlas
GEPIA: Gene Expression Profiling Interactive Analysis
BRAF: B Raf Proto-oncogene Serine/Threonine Kinase
TLR9: Toll-Like Receptor 9
CDK3: Cyclin-Dependent Kinase 3
PDK1: Pyruvate Dehydrogenase Kinase Isoenzyme 1
RBM43: RNA-Binding Motif Protein 43
CYP: Cytochrome P450
