# Supplementary material for: Bioinformatics screening the novel and promising targets of curcumin in hepatocellular carcinoma chemotherapy and prognosis
Source: BMC Complement Med Ther. 2022 Jan 25;22:21. doi: 10.1186/s12906-021-03487-9 (PMC8788085; doi:10.1186/s12906-021-03487-9)
Supplement: Supplementary file 1 — Additional file 1: Supplemental Table 1. Simple parameters of PPI network analysis. Supplemental Table 2. The top list of hub nodes in PPI network (Degree distribution≥12). [file 12906_2021_3487_MOESM1_ESM.docx]

**Supplemental Table 1.** Simple parameters of PPI network analysis

| Clustering coefficient | 0.534 | Number of nodes | 105 |
| --- | --- | --- | --- |
| Connected component | 1 | Network density | 0.111 |
| Network diameter | 6 | Network heterogeneity | 0.836 |
| Network radius | 3 | Isolated nodes | 0 |
| Network centralization | 0.417 | Number of self-loops | 0 |
| Shortest paths | 10920(100%) | Multi-edge node pairs | 0 |
| Characteristic path length | 2.415 | Analysis times(sec) | 0.029 |
| Avg. number of neighbors | 11.505 |  |  |

**Supplemental Table 2.** The top list of hub nodes in PPI network (Degree distribution≥12)

| Name | Degree | Betweenness  Centrality | Stress | Neighborhood  Connectivity | MCODE Score |
| --- | --- | --- | --- | --- | --- |
| AKT1 | 54 | 0.17461482 | 10962 | 14.87037037 | 9.220588235 |
| EGFR | 50 | 0.18565112 | 12134 | 15.22 | 8.076923077 |
| STAT3 | 38 | 0.07507717 | 6306 | 17.44736842 | 7.714285714 |
| HSP90AA1 | 36 | 0.08312047 | 5698 | 17.02777778 | 6.368421053 |
| MMP9 | 35 | 0.08035381 | 5816 | 16.91428571 | 8.509090909 |
| EP300 | 27 | 0.0436099 | 3030 | 20.25925926 | 9.638095238 |
| CDK1 | 26 | 0.03424955 | 2848 | 21 | 11.73626374 |
| ABL1 | 26 | 0.05181213 | 4212 | 18.5 | 9 |
| PPARG | 25 | 0.06105833 | 3716 | 17.12 | 3.828571429 |
| CDK4 | 24 | 0.01375964 | 1430 | 21.79166667 | 11.73626374 |
| CDK2 | 23 | 0.00885367 | 1456 | 22 | 11.73626374 |
| CCNA2 | 23 | 0.00931676 | 1920 | 23.2173913 | 11.73626374 |
| CHEK1 | 23 | 0.00929962 | 1516 | 22.08695652 | 11.73626374 |
| CCNB1 | 22 | 0.0081208 | 1542 | 23.77272727 | 11.73626374 |
| E2F1 | 21 | 0.01236741 | 1476 | 22.95238095 | 11.73626374 |
| CCNE1 | 19 | 0.00491599 | 1198 | 23.68421053 | 11.73626374 |
| AURKA | 19 | 0.00532601 | 1074 | 23.21052632 | 9.463235294 |
| F2 | 19 | 0.0475986 | 3148 | 17.05263158 | 6.611111111 |
| TOP2A | 17 | 0.00230257 | 550 | 21.70588235 | 10.85897436 |
| APP | 17 | 0.02178213 | 1984 | 20.82352941 | 5.785714286 |
| SERPINE1 | 17 | 0.01551331 | 970 | 18.58823529 | 5.515151515 |
| AURKB | 16 | 0.00210088 | 352 | 20 | 11.73626374 |
| CYP3A4 | 16 | 0.07609935 | 4484 | 12 | 6 |
| GSK3B | 15 | 0.00385747 | 468 | 22.93333333 | 4.307692308 |
| WEE1 | 15 | 0.00163226 | 464 | 25.46666667 | 10.85897436 |
| CCNA1 | 15 | 0.0022953 | 102 | 18.26666667 | 11.73626374 |
| RPS6KB1 | 15 | 0.0024125 | 276 | 26.66666667 | 8.509090909 |
| EPHA2 | 15 | 0.01736854 | 1922 | 22.26666667 | 9 |
| ABCG2 | 15 | 0.04234003 | 3228 | 19.4 | 3.03030303 |
| EPHB2 | 14 | 0.00764824 | 704 | 16.28571429 | 9 |
| GSTP1 | 14 | 0.02292476 | 2744 | 18.42857143 | 6 |
| CDK3 | 13 | 2.19E-04 | 70 | 21.69230769 | 10.85897436 |
| CYP2B6 | 13 | 0.02083858 | 1726 | 11.84615385 | 6 |
| NFE2L2 | 13 | 0.02975784 | 2594 | 21.07692308 | 4.166666667 |
| TOP1 | 12 | 0.00269038 | 334 | 27.08333333 | 6.109090909 |
| CYP2C9 | 12 | 0.0117911 | 914 | 10.58333333 | 6 |
| MMP13 | 12 | 0.00425378 | 406 | 22.08333333 | 7.466666667 |
| CXCR2 | 12 | 0.01017571 | 978 | 22.41666667 | 6 |
| ADAM17 | 12 | 0.00466996 | 470 | 21.75 | 6.611111111 |
